# Perioperative Medicine for Older People Undergoing Surgery Scale Up (POPS-SUp): study protocol

**DOI:** 10.1093/bjsopen/zraf063

**Published:** 2025-11-19

**Authors:** Jugdeep K Dhesi, Judith S L Partridge, Bridget C Strasser, Lindsay Bearne, Nathan Hall, Andrew Healey, John S M Houghton, Laura Magill, Bijan Modarai, Iain K Moppett, Lawrence Mudford, John Norrie, Rupert M Pearse, Thomas Pinkney, Athanasios Saratzis, Robert Sayers, Cecilia Vindrola-Padros, Justin Waring

**Affiliations:** Perioperative Medicine for Older People Undergoing Surgery (POPS), Guy’s and St Thomas’ NHS Foundation Trust, London, UK; Ageing Research, Population Health Sciences, Life Sciences and Medicine, King’s College London, London, UK; Perioperative Medicine for Older People Undergoing Surgery (POPS), Guy’s and St Thomas’ NHS Foundation Trust, London, UK; Ageing Research, Population Health Sciences, Life Sciences and Medicine, King’s College London, London, UK; Perioperative Medicine for Older People Undergoing Surgery (POPS), Guy’s and St Thomas’ NHS Foundation Trust, London, UK; Ageing Research, Population Health Sciences, Life Sciences and Medicine, King’s College London, London, UK; Population Health Research Institute, City St George’s, University of London, London, UK; NHS England, London, UK; Health Services and Population Research Department, Institute of Psychiatry, Psychology and Neuroscience, King’s College London, London, UK; University of Leicester, Department of Cardiovascular Sciences, NIHR Leicester Biomedical Research Centre, Leicester, UK; Birmingham Centre for Observational and Prospective Studies (BiCOPS), University of Birmingham, Birmingham, UK; Cardiovascular and Metabolic Medicine and Sciences, British Heart Foundation Centre, Life Sciences and Medicine, King’s College London, London, UK; Anaesthesia and Perioperative Medicine, University of Nottingham, Nottingham, UK; Nottingham University Hospitals NHS Trust, Nottingham, UK; Centre for Perioperative Care (CPOC), London, UK; School of Medicine, Dentistry and Biomedical Sciences, Queen’s University, Belfast, Ireland; Critical Care and Perioperative Medicine , Queen Mary University of London, London, UK; Birmingham Centre for Observational and Prospective Studies (BiCOPS), University of Birmingham, Birmingham, UK; University of Leicester, Department of Cardiovascular Sciences, NIHR Leicester Biomedical Research Centre, Leicester, UK; University of Leicester, Department of Cardiovascular Sciences, NIHR Leicester Biomedical Research Centre, Leicester, UK; Department of Targeted Intervention, University College London, London, UK; School of Social Sciences, Loughborough University, Loughborough, UK

## Abstract

**Background:**

Surgery provides definitive management of many age-related diseases, relieving symptoms or extending life. Age-related physiological decline, multimorbidity, and frailty predispose older people to postoperative complications and incomplete functional recovery, with resultant health and social care costs. These age-related conditions can be optimized using Comprehensive Geriatric Assessment (CGA), thus mitigating perioperative risk to improve clinical outcomes with cost-effectiveness. National organizations advocate CGA-based services for older surgical patients. However, there is variation in the provision of CGA-based perioperative medicine for older people undergoing surgery (POPS) services across the UK National Health Service, resulting in inequitable access for older surgical patients at higher risk, unnecessary deaths, complications, and financial cost. The aim of the POPS Scale Up (POPS-SUp) study is to determine whether CGA-based POPS services can be implemented at scale to cost-effectively improve clinical outcomes for older patients undergoing surgery.

**Methods:**

A mixed-methods hybrid implementation–effectiveness interrupted time series study will examine the use of a coproduced implementation strategy to embed CGA-based POPS services at scale in the UK. Co-primary implementation–effectiveness outcomes will be used, namely reach and length of hospital stay, respectively. Evaluation will include an embedded process evaluation, quantitative evaluation of clinical effectiveness and cost-effectiveness, and qualitative appraisal of patient and staff experience. The proposed analysis is to embed a process evaluation using real-time framework analysis, enabling iterative refinement and evaluation of the implementation strategy. Accepted interrupted time series analysis will be used to examine and compare outcomes per participating site. A predefined dissemination strategy has been co-designed with patients/carers, clinical community of practice, and organizational bodies.

**Conclusion:**

The anticipation is that POPS-SUp will have impact at the individual (patient and clinician), organizational, and policy levels in the perioperative setting, but with additional potential application to other clinical settings.

Registration numbers: ISRCTN 45327 (https://www.isrctn.com/); NIHR 157443 (https://www.nihr.ac.uk/).

## Teaser

The number of older people undergoing surgery is increasing and age-related physiological decline, multimorbidity, frailty, and dementia predispose older people to postoperative complications and incomplete functional recovery, with resultant health and social care costs. Perioperative Medicine for Older People Undergoing Surgery Scale Up (POPS-SUp) is a hybrid implementation–effectiveness interrupted time series study using mixed methodology to examine whether comprehensive geriatric assessment and optimization based perioperative medicine services can be implemented at scale to cost-effectively improve clinical outcomes for older patients undergoing elective and emergency surgery. A predefined dissemination strategy has been co-designed with patients, carers, clinical community of practice, and organizational bodies.

## Introduction

Two-thirds of elective and two-fifths of emergency surgical procedures in the UK National Health Service (NHS) are undertaken in people aged over 65 years^1^. Surgery offers definitive management of many age-related diseases, relieving symptoms and extending life. These benefits are weighed against the risk of adverse outcomes. Age-related physiological decline, multimorbidity, frailty, and dementia predispose older people to postoperative medical complications such as pneumonia, acute kidney injury, and delirium^2,3^. Such complications result in higher postoperative mortality, slower and incomplete functional recovery, poorer experience, and a higher use of NHS resources among older people^4^. Predisposing age-related conditions can be identified and modified using comprehensive geriatric assessment (CGA) and optimization. This holistic process involves clinical skills and standardized tools to assess medical, functional, psychological, and social domains, prompting individualized evidence-based interventions. Examples include preoperative optimization of hypertension or anaemia, medication modification to reduce delirium or falls, better-informed shared decision making, the anticipation and provision of rehabilitation, and early home adaptations^5^. Level 1 evidence demonstrates that older people receiving CGA during an acute medical admission are more likely to be alive and living at home at 1 year^6^. Similarly, CGA cost-effectively reduces morbidity and mortality after elective or emergency surgery, increasing the likelihood of discharge home^7–10^. Within the UK, national organizations such as the Royal College of Surgeons of England, Department of Health, National Emergency Laparotomy Audit, and Centre of Perioperative Care advocate CGA-based services for older patients undergoing elective and emergency surgery^11–13^.

**Fig. 1:**
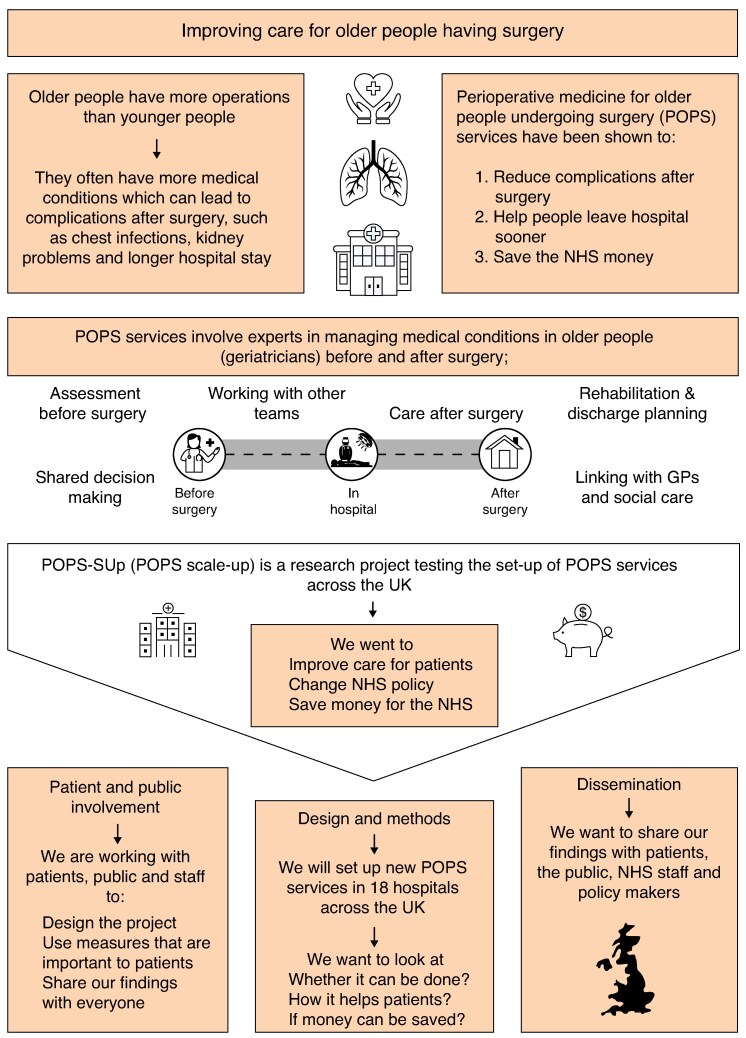
Perioperative Medicine for Older People Undergoing Surgery Scale Up (POPS-SUp) infographic NHS, National Health Service; GPs, general practitioners.

Although some hospitals have established CGA-based perioperative medicine for older people undergoing surgery (POPS) services, others have been unsuccessful^14^. Reported barriers to establishing POPS services include inadequate funding and workforce and a lack of interspeciality collaboration^14^. This has resulted in unacceptable variation in access to quality care for high-risk, older surgical patients across the NHS, leading to unnecessary deaths, complications, and excess NHS costs^13,15^. The challenges to systematic scale up of complex interventions (such as POPS services) include a heterogeneous patient population, multiprofessional stakeholders, the need to ensure fidelity to the intended intervention, and failure to adapt the intervention to the local context^16^. Overcoming barriers to NHS scale up of complex interventions, such as POPS services, requires a systematically co-designed implementation strategy^16,17^, as illustrated by the successful translation of the POPS model using a logic model approach (*Table S1*)^18^. The Perioperative Medicine for Older People Undergoing Surgery Scale Up (POPS-SUp) study will investigate two interlinked interventions: first, an implementation strategy designed to support implementation of POPS services; and second, the clinical effectiveness and cost-effectiveness of POPS services established using that implementation strategy to deliver perioperative CGA-based care (*Fig. 1*).

## Methods

### Research question

The research question being addressed in the POPS-SUp study is whether CGA-based perioperative medicine services (POPS services) can be implemented throughout the NHS to cost-effectively improve clinical outcomes for older patients undergoing elective and emergency surgery.

### Objectives

The POPS-SUp programme of work will examine the clinical effectiveness and cost-effectiveness of CGA-based POPS services at scale in the perioperative NHS setting, as well as the impact of a co-designed iterative implementation strategy to scale up a complex intervention (CGA-based POPS service).

### Study design

A hybrid implementation–effectiveness interrupted time series (ITS) study will examine whether CGA-based perioperative medicine services (POPS) can be implemented throughout the NHS to improve clinical outcomes for older patients undergoing elective or emergency surgery with cost-effectiveness for the NHS.

### Study interventions

POPS-SUp will examine two inter-linked interventions. The first intervention is a trimodal implementation strategy designed to support implementation of POPS services; the second intervention is CGA-based care delivered by the POPS service.

### Implementation strategy

**Fig. 2:**
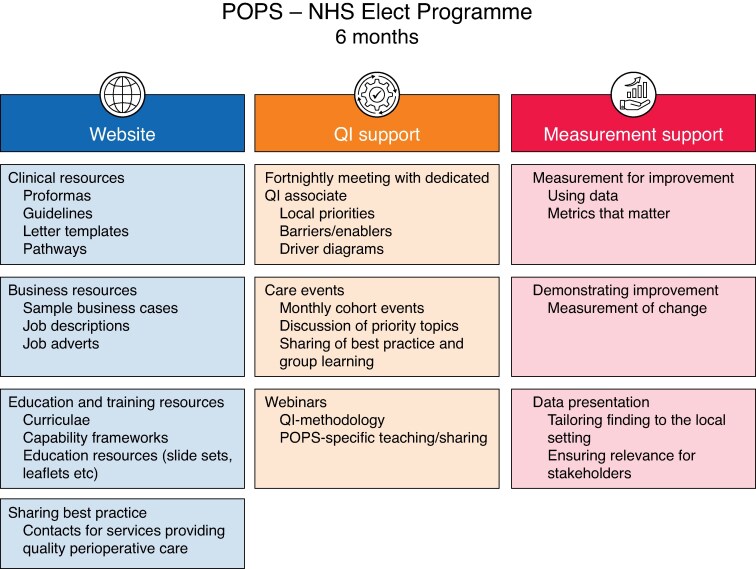
Trimodal implementation strategy delivered through the NHS elect programme POPS, Perioperative Medicine for Older People Undergoing Surgery; NHS, National Health Service; QI, quality improvement.

The trimodal POPS implementation strategy uses a toolkit, quality improvement coaching and mentoring, and training in the use of data and measurement to deliver improvement. The toolkit includes clinical resources, education and training, and business resources (*Fig. 2*). This implementation strategy was co-produced with patients and carers, as well as clinical, academic, and managerial stakeholders.

NHS Elect is a national NHS organization that helps individuals, teams, and organizations in health and care to improve the quality of care they deliver by providing training; personal, team and organizational development; and consultancy services. NHS Elect will support the delivery of this implementation strategy through structured online meetings between participating hospital site teams and expert coaches (with expertise in clinical POPS services, improvement science, and data management). The NHS Elect POPS programme will include an initial site visit, team meetings every 2 weeks, monthly events for the cohort, and regular webinars. The POPS-SUp study will evaluate the impact of this co-produced implementation strategy on implementation outcomes.

### Perioperative CGA-based care delivered through a POPS service

**Fig. 3:**
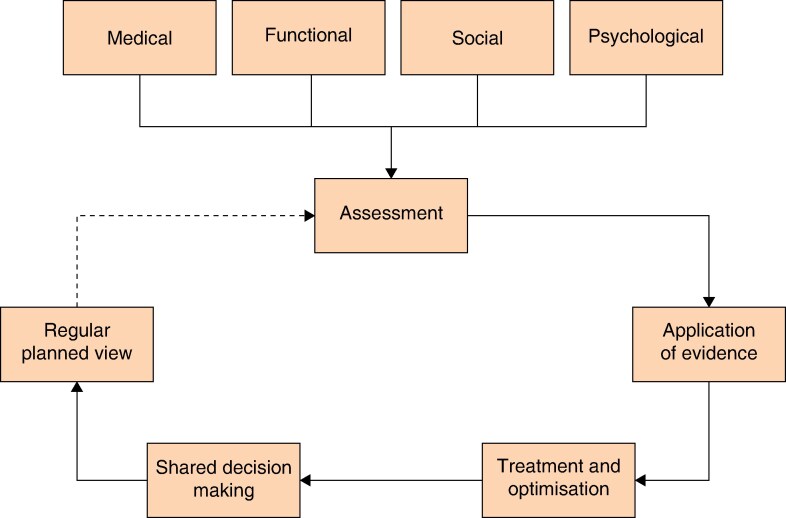
Comprehensive geriatric assessment and optimization

The POPS services to be evaluated in the POPS-SUp study use comprehensive geriatric assessment and optimization (CGA) methodology (*Fig. 3*). CGA involves the holistic assessment of a patient across medical, functional, social, and psychological domains, using objective measures to inform multidisciplinary optimization. The POPS service, using CGA methodology at each of the 18 participating hospitals, will be delivered by a geriatrician-led multidisciplinary team from that hospital, supported by the trimodal NHS Elect POPS implementation strategy. Patients under the care of general and/or orthopaedic and/or urological and/or vascular surgery teams at all study sites will receive perioperative care delivered through the planned intervention, namely the POPS service implemented through the trimodal implementation strategy, supported by the NHS Elect POPS programme. The POPS service will deliver perioperative care for patients living with frailty, multimorbidity, cognitive issues, and/or those in whom the decision to operate is not clear, who are being considered for major emergency and/or elective general and/or orthopaedic and/or urological and/or vascular surgery. Patients undergoing hip fracture surgery are deliberately excluded due to well established pathways of orthogeriatric care in the UK.

### Setting

Eighteen NHS hospitals (two sequential cohorts of eight to ten hospitals) providing general and/or orthopaedic and/or urological and/or vascular surgery located across England, Scotland, Wales and Northern Ireland will be included in the study. These hospitals will be recruited through contact from the chief investigator and sampled purposively to ensure variations in the type and size of hospitals (district general/teaching hospital), geographical location (rural/urban), patient populations served (ensuring ethnic and socioeconomic diversity), and types of surgery undertaken (ensuring inclusion of elective and emergency surgery). These sites will be required to have a geriatrician (consultant, speciality, and specialist doctors) with allocated time to support implementation and support from a hospital executive board member.

### Participants

There will be three groups of study participants at each hospital site, namely NHS staff implementing the POPS services, patients aged ≥50 years, and carers/family members of patients aged ≥50 years. Specific inclusion and exclusion criteria are listed below.

Inclusion criteria:

- NHS staff implementing POPS services, including geriatricians, anaesthetists, surgeons, nursing and allied health professionals, and managers
- patients aged ≥50 years under the care of general and/or orthopaedic and/or urological and/or vascular surgeons: a non-consented group in whom routine clinical data will be recorded and a consented group who will complete questionnaires and interviews (the inclusion of those aged > 50 years was chosen to ensure that those living with multiple long-term health conditions or frailty were recruited)
- carers/family members closely involved in the care of patients aged > 50 years under the care of general and/or orthopaedic and/or urological and/or vascular surgeons; some of these carers/family members will be related to patient participants recruited as per the criteria above, whereas others may be related to patients who do not display capacity to consent to the study.

Exclusion criteria:

- at NHS staff level: none
- at patient participant level: non-consenting, patients who request to ‘opt out’ and prisoners; consenting, patients with mental or cognitive impairment so severe as to preclude completion of the 9-item Shared Decision Making Questionnaire^19^ (SDM-Q-9) and/or the Decisional Regret Scale^20^ (DRS) and/or no capacity to consent to study and prisoners
- at carer/family member level: carer/family member who is only distantly involved in the care of patients aged ≥50 years under the care of general and/or orthopaedic and/or urological and/or vascular surgeons.

### Sampling and recruitment

#### Staff participants

Principal investigators at each participating site will identify NHS staff involved in the implementation and delivery of the CGA-based POPS service. These staff will be informed about the study through posters and leaflets. For the ethnographic component of the POPS-SUp study where researchers observe the practical delivery of the POPS service, staff who do not wish to be observed in the workplace will be given the opportunity to opt out. Staff who wish to participate in the interview component of the POPS-SUp study will be provided with a participant information sheet and the opportunity to ask questions before providing written consent for study participation.

#### Patient participants

At each participating hospital site, the clinical inclusion criteria (patients who will be managed by the POPS intervention) will be defined with the NHS Elect team and chief investigator. These criteria will be based on the surgical area in which the POPS service is being implemented. For example, one site may recruit patients aged > 60 years being considered for elective aortic aneurysm repair, another may recruit patients aged > 70 years presenting to the emergency general surgeons, and another may recruit elective and emergency patients aged > 65 years undergoing bladder and prostate surgery. At each hospital site, there will be two groups of patient participants: non-consented and consented.

For non-consenting patients, routinely collected clinical data from patients meeting the inclusion criteria will be recorded with Health Research Authority Confidentiality Advisory Group, NHS Scotland Public Benefit and Privacy Panel for Health, and Northern Ireland Information Governance approval. At each site, the clinical and research team will ensure that visibility of the POPS-SUp study is promoted in all clinical areas using posters and leaflets and that patients are aware they are free to opt-out. All other patients meeting the inclusion and exclusion criteria at each site will have routinely collected clinical data (*Table S2*) recorded in the study database.

For consented patients, a purposive sampling strategy will aim to recruit patients with a range of experiences of the surgical pathway, with clinical teams identifying patients with a short and long length of hospital stay, those with and without postoperative complications, and those undergoing surgery or conservative management. Patients will be provided with a participant information sheet and the opportunity to ask questions before providing written consent for study participation.

#### Carer participants

The POPS-SUp study will purposively sample carers/family members who are closely and regularly involved in the care of patients meeting the inclusion and exclusion criteria, including where those patients have cognitive impairment, dementia, or delirium impairing capacity to consent. Carers/family members will be provided with a participant information sheet and the opportunity to ask questions before providing written consent for study participation.

### Recruitment targets

The recruitment targets for NHS staff and consenting and non-consenting patients are as follows:

- NHS staff: expected recruitment rate five to ten members of staff per site (total 114)
- non-consented patient participants: expected patient recruitment rate 10–15 patients per month per site; each site will contribute 120–180 patients over 12 months (total 2500)
- consenting patient participants: expected patient recruitment rate six patients before implementation and six patients after implementation at each site (total 216); over a 12-month period, the nine sites are expected to contribute 1080–1620 patients, and over the two periods (the total for the study) it is expected the sites will contribute 2160–3240 patients.

### Outcomes

Primary and secondary outcomes for the POPS-SUp study are presented in *Table 1*.

**Table 1:** Primary and secondary outcomes

Primary outcomes	
Implementation outcome	Reach (%)
	Calculated as number of patients seen by POPS services/number of patients eligible for POPS review
	Predefined according to which surgical speciality the service is being established in
Clinical outcome	Length of hospital stay (days)
**Secondary outcomes**	
Implementation outcome	Fidelity to clinical components of perioperative CGA
	Case note review of all patients seen by the POPS services to establish fidelity to the core components checklist for CGA
	Fidelity to core components of POPS services
	Measured against POPS logic model core components (see appendix 1 in Brehaut *et al*.^20^), which will be adjusted according to the service being established Measured through process evaluation (staff members being interviewed/observed/surveyed)
	Acceptability and feasibility of the implementation strategy
	Assessed through process evaluation
Clinical outcome	30-day readmission (HES linkage)
	Postoperative complications according to predefined characteristics (see *Table 1*: routinely collected clinical data)
	Same-day cancellation
	Return to preoperative place of residence (clinical record)
	Days alive and out of hospital 90 days (HES linkage)
	90-day and 12-month mortality (HES linkage)
	Operative or non-operative management
	Was the initially suggested procedure undertaken or did the patient undergo a different or no procedure?
	Clinician defined, ‘medically fit for discharge’
	Notes review at or after discharge
	Shared decision making (SDM-Q-9) collected in a purposively sampled consented subgroup of patients
	Collected in the preimplementation and postimplementation phases
	Decisional regret (DRS) collected in a purposively sampled consented subgroup of patients
	Collected in the preimplementation and postimplementation phases

POPS, perioperative medicine for older people undergoing surgery; CGA, comprehensive geriatric assessment; HES, Hospital Episodes Statistics; SDM-Q-9, 9-item Shared Decision Making Questionnaire; DRS, Decision Regret Scale.

### Data collection

**Fig. 4:**
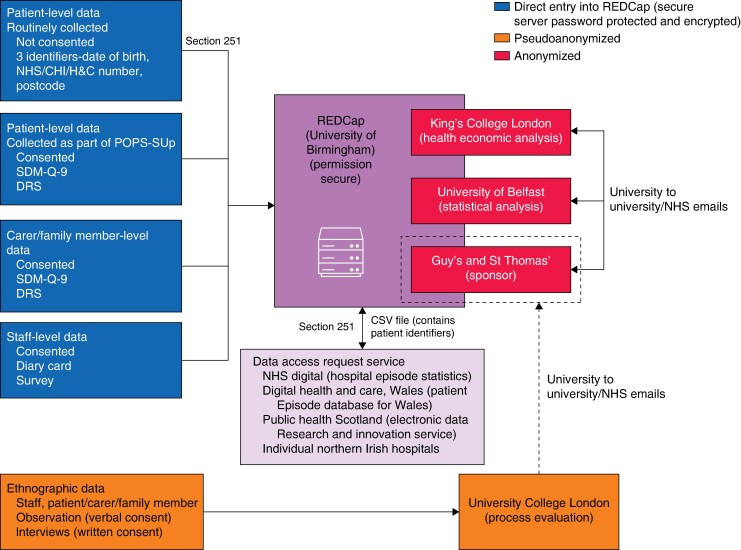
Data flow diagram NHS, National Health Service; CHI, Community Health Index; H&C, Health and Care; SDM-Q-9, 9-item Shared Decision Making Questionnaire; DRS, Decision Regret Scale; POPS-SUp, Perioperative Medicine for Older People Undergoing Surgery Scale Up.

Data will be collected as outlined in *Table S2* and entered in the REDCap database (*Fig. 4*).

#### Staff

Staff will self-complete diary cards indicating the time spent participating in POPS implementation and the time spent delivering patient care through the CGA-based POPS service. This information will be used for health economic analysis.

Ethnographic observation of staff undertaking both implementation activities and clinical activities will be undertaken by the research team.

In a consenting subgroup of staff semistructured, recorded, and transcribed interviews will be undertaken by a researcher and analysed according to the Consolidated Framework for Implementation Research.

Ethnographic and interview data will be used to report fidelity to the implementation programme and to the process of perioperative CGA.

#### Patients (non-consented)

Patient-level clinical data from each site will be entered directly into a secure web-based application by each local research team. Secondary clinical outcomes, including 90-day and 12-month mortality, readmissions within 30 days, and days alive and out of hospital at 90 days, will be obtained through data linkage with NHS digital for Hospital Episode Statistics and the Office for National Statistics.

#### Patients and carers (consenting)

Consenting patients will participate in semistructured interviews, which will be recorded and transcribed by the research team, and will be helped to complete to questionnaires (SDM-Q-9 and DRS, with the latter to be used for health economic analysis; see *Fig. 4*).

### Data analysis

#### Sample size

The sample size has been calculated using the primary clinical outcomes measure of length of hospital stay (days). An unweighted one-sample *t* test with each of 18 sites contributing a single after–before data point would have 90% power at the 5% level of significance to detect an effect size of 0.8 that is a difference in the mean length of stay after/before implementation of 0.8 standard deviations. If the standard deviation at the site level was 5 days, this would equate to being able to detect a mean difference of 4 days, which would be sufficient to impact clinical practice.

The true power of the study is estimated to be greater (for example, allowing the detection of a smaller mean difference of 2–3 days), and will be accurately assessed using simulation informed by early data from the study, and using the statistical model to be employed, which will depend on individual-level data within each site, potentially at multiple time points (for example, each week for around 12 times in each of the 3-month before and after periods), and fully account for dependency across participants within sites, and adjusting for known prognostic factors.

#### Statistical analyses

Analysis of the ITS will adopt accepted recommendations providing flexibility to model evolving variability and correlation between the before and after periods^21–23^.

The primary analysis will include all participants recruited to the study where possible (akin to an intention-to-treat analysis) consistent with a treatment policy estimate^24,25^.

There will be two cohorts of nine hospital sites recruited (*n* = 18), with data collection occurring over 12 months in each cohort (3 months of data collection before implementation, 6 months during implementation, and 3 months after implementation).

The statistical analysis plan covers the clinical effectiveness of this hybrid implementation–effectiveness study. Compliance with the intervention and/or the fidelity of intervention implementation is measured and assessed separately through embedded process evaluation. There will not be any statistical modelling (for example, causal effect modelling) to adjust the treatment effects for any measure of compliance.

Categorical data will be presented using counts and percentages, whereas continuous variables will be presented using the mean, median, standard deviation, interquartile range, and number of patients with an observation.

Box plots will be produced that are graphical summaries of the distribution, including mean, median, first and third quartiles, minimum and maximum values, for before and after the intervention, and by site and period.

Measurements will be made on an individual basis as the total length of hospital stay, including any readmission within 30 days of discharge.

The treatment response will be estimated in anticipation that the shape of the treatment response will incorporate a gradual change in the slope (trend) and a gradual change in the ‘step’ (intercept), and that there may be a delay (lag) in either or both of these effects (slope and intercept)^21–23^.

A times series model will be used with a continuous outcome with appropriate transformation as necessary to address skewness, with estimates and 95% confidence intervals back-transformed to the original untransformed scale of days^26,27^.

The model will estimate the effect of the intervention on the co-primary outcome of (untransformed or transformed) length of hospital stay, with terms for treatment (after—before periods, within site) accounting for any deaths, and adjusting for local site effects (including staggered times of intervention) and any temporal trends (potentially non-linear) and adjust for either site- or individual-level co-variables strongly related to the outcome.

All the assumptions regarding the statistical model will be assessed, including autocorrelation structure and non-stationarity, as well as seasonality, if appropriate.

The length of hospital stay will be measured in days at the individual level and analysed at the site level. Reach will be measured as a proportion, calculated as the number of patients receiving the intervention divided by the number of patients eligible to receive the intervention.

The secondary outcomes will be analysed in a similar way to the primary outcome, with a statistical model appropriate for the specific secondary outcome (for example binary or ordinal logistic regression, time-to-event (Cox) regression, linear regression).

Predefined subgroup analysis will be restricted to the primary clinical effectiveness outcome alone. Any further subgroup analysis (for example, if suggested later by new data external to the study) will be labelled exploratory. Prespecified subgroup analyses will be unlikely to be adequately powered.

It is not anticipated that there will be many missing data for the primary outcome (length of hospital stay). Nonetheless, the robustness of the findings to any patterns of missing data will be checked using sensitivity analyses (including multiple imputation under an assumption of missing at random, or possibly pattern mixture type models for informative missingness)^28^.

A multiple imputation approach will be used assuming the data are missing at random. In addition, and probably more consistent with the likely missing data-generating mechanisms, sensitivity-type analyses assuming the data are missing not at random (that is, informatively missing) will be explored using pattern mixture models, or tipping point-type approaches. These sensitivity analyses would attempt to identify different types of missing data by an underlying reason or reasons, and then impute values that capture plausible measurements for those missing data. The γ-adjustment approach^29^ will be followed, as will the recommendations on sensitivity analyses^30^.

The safety data (for example, medical and surgical complications, factors around delayed discharge, delirium, acute coronary syndrome, cardiac failure, arrhythmias, pneumonia, wound infection, urinary tract infections, faecal incontinence, falls, acute postoperative complications (cardiac, pulmonary, infections, bowel/bladder, vascular), level 2/3 care after surgery, and other adverse events) will be presented descriptively.

This analysis plan describes the end-of-trial statistical analyses to be performed for the POPS-SUp study. There will be no formal interim analyses.

There will be a sample size check/re-estimation step at or around the end of the first cohort of nine sites followed for the first 12 months, which will validate the assumptions behind the power calculation (specifically the assumed common standard deviation) and, in particular, upgrade the estimation of the actual power of the study using simulation, using the appropriate statistical model for the primary outcome of length of stay, instead of the simple approximation using a one-sample *t* test as above. The timing of the sample size re-estimation coincides with the end of the first period of 12 months. See Zhang *et al.*^31^ and Hawley *et al.*^32^ for further details on sample size estimation in ITS designs.

All analysis and data manipulation will be performed using SAS, R or Stata unless otherwise stated^33–35^.

A file, or set of files, containing the final data will be prepared, along with a data dictionary. These will be made available to the chief investigator at the end of the analysis phase (*Fig. 4*).

#### Health economic plan

A cost–consequences analysis (CCA) will be undertaken. This will estimate the incremental cost of implementing and delivering the intervention (CGA-based POPS services) within routine care at scale, and the incremental effect of the intervention on perioperative costs. Estimates of the overall incremental intervention cost (implementation and service delivery cost minus postoperative care costs) will be presented alongside wider intervention consequences for patients and staff being explored within the main statistical analysis and process evaluation. Analysis of the cost impact of the intervention will be undertaken using a cohort-based decision modelling approach informed by similar previous work^8^. Parameters in the model will include the expected proportion of patients exposed to surgery (for an elective patient cohort) and the expected proportion of those undergoing surgery who experience postoperative complications after implementation of a POPS service compared with standard (before implementation) practice. The model will also include the postoperative costs associated with these outcomes, including a consideration of costs relating to the length of hospital stay, postoperative contact with healthcare professionals, the use of medical consumables, time spent in critical care, discharge destination, and the cost of readmissions. Parameter values and uncertainty arising from sampling error will be incorporated into the model through statistical analysis of patient-level data extracted from study sites over the before and after implementation periods. A secondary cost–utility analysis will also be undertaken to assess the incremental cost per quality-adjusted life-years gained of implementing POPS services within defined patient populations^36^. This will enable benchmarking against existing thresholds for determining value for money in the NHS. All economic analyses will be undertaken from an NHS/personal social services perspective in line with the National Institute for Heath and Care Excellence reference case^37^. The following data sources from across the study sites will support the economic evaluation:

- Healthcare professional activity logs completed across all 18 study sites by a sample of staff with reference to a specified period, recording time and resources allocated to implementation and delivery of POPS at scale. Comparable data will also be collected during the preimplementation period to enable a baseline estimate of assessment-related costs without POPS.
- Clinical case notes data across all 18 study sites recording: patient contact with healthcare professionals and the use of healthcare resources before and after surgery, as well as after discharge from hospital; preoperative clinical and demographic data; and postoperative medical complications, including mortality.

#### Process evaluation

The process evaluation will use a formative design with findings regularly shared with the implementation team to inform implementation. Rapid analysis techniques (where data are analysed in parallel with data collection) and feedback loops will be used to facilitate the sharing of emerging findings^38^. Framework analysis will be used to perform more in-depth analysis, bringing together the interview, observational, and documentary data to create individual case studies and explore variation in implementation^39^. The framework will be informed by the Consolidated Framework for Implementation Research, but the researchers will also be sensitive to new topics generated from the data^40^.

### Patient and public involvement and engagement

The POPS-SUp team has conducted extensive patient and public involvement and engagement (PPIE) work in the piloting and set up of POPS-SUp that informed the funding application and design of the study. This PPIE work has been undertaken with three groups: patients and the public; community of practice; and supporting organizations (*Table 2*).

**Table 2:** Patient and public involvement and engagement

PPIE 1: Patients and their carers/family members	
Public advisory group	Eight to ten patients/carers with lived experience of different sex, from ethnically diverse communities, and localities
**PPIE 2: Community of practice**	
Supporting sites	Addenbrookes Cambridge University Hospital
	Darent Valley Hospital
	Craigavon Area Hospital, Southern Health and Social Care Trust
	Frimley Park Hospital
	King’s College Hospital NHS Foundation Trust
	Lewisham and Greenwich NHS Trust
	Northwick Park Hospital
	Queen Victoria Hospital
	Royal Derby Hospital
	Royal Devon Hospital
	Royal Surrey County Hospital
	Swansea Bay University Health Board
	The Dudley Group NHS Foundation Trust
	University College London Hospital NHS Trust
	University Hospital of Wales, Cardiff
	University Hospital Sussex NHS Foundation Trust
	Whipps Cross Hospital
	Wirral University Hospital NHS Trust
**PPIE 3: Professional stakeholder organizations**	
Healthcare professional organizations	Association of Anaesthetics
	Association of Surgeons of Great Britain and Ireland
	British Geriatric Society
	Centre of Perioperative Care
	Perioperative Quality Improvement Program
	Vascular Anaesthesia Society of Great Britain and Ireland
	Vascular and Endovascular Research Network
	Vascular Society
Charities/patient advocacy groups	Age UK
	Independent Age
	Patient Information Forum
	South Asian Health Action
Policy makers/service planners	Centre for Research Equity
	Getting It Right First Time
	Human Sciences Research Council National Institute of Academic Anaesthesia
	National Emergency Laparotomy Audit
	NHS Improvement and NHS England
	National Institute of Health Research Ageing and Clinical Research Network
	The Healthcare Improvement Studies Institute

PPIE, patient and public involvement and engagement.

All three groups have expressed an urgency to undertake the POPS-SUp study, which, they believe, will address many of the current challenges in perioperative care. In particular, patients told us POPS services will help them prepare for surgery, support them with decision making, and help them recover from surgery more quickly and in their preferred place. The community of practice described the appetite for POPS services among clinicians, and supporting organizations emphasised the need to scale up POPS services at pace to reduce geographical variation in perioperative care for older patients.

Patient and public involvement and engagement continues to be central to POPS-SUp. The POPS-SUp team will continue to work with the three advisory groups and ensure diversity and equality of opportunity, enabling everyone to be actively involved in this research. The study will draw on National Institute for Health and Care Research (NIHR) definitions, where inclusion means taking deliberate action to meet the needs of different people and promoting environments where everyone is respected, valued for who they are, and able to achieve their full potential. NIHR defines diversity as understanding everyone is unique, respecting and valuing all forms of difference^41^.

The patient and public advisory group and community of practice will meet twice a year and provide ad hoc input to develop research methods and materials, maximizing acceptability, participation, inclusion, and knowledge mobilization. Two public advisors with lived experience will contribute to study management and independent oversight committee with allocated budget. Induction, training, support, and coaching will be offered. Effective communication between stakeholder groups and the research team is fundamental to the success of this project. The accountable academic lead for PPIE and the public advisor will be responsible for ensuring strong links are maintained between the stakeholder groups and research team.

An appropriate budget has been included in this bid to support all stakeholder and public involvement and engagement activities, including dissemination.

### Study governance

The study has received regulatory approval from the Health Research Authority Approval (HRA) 335587, Confidentiality Advisory Group (33587), and NHS Scotland Public Benefit and Privacy Panel for Health and Social Care.

All correspondence with the Sponsor, REC, and HRA will be retained. An independent study steering committee will oversee the conduct of the study and meet twice yearly.

A stakeholder mapping exercise has been conducted to identify all relevant stakeholders for dissemination and maximal impact. Study updates and findings will be shared, using various formats (academic publication, conference presentation, social media, lay summary, press releases and reports, networking events, infographics, websites) as appropriate for each audience, including: patient and carer participants, clinical and research teams, and NHS chief executives at participating hospitals; clinicians and academics from all relevant disciplines; healthcare policymakers locally and nationally; NICE; healthcare professional organizations; and charities and patient-facing organizations.

Academic publications will be reported according to Standard Protocol Items: Recommendations for Interventional Trials (SPIRIT; for protocol)^42^, Consolidated Health Economic Evaluation Reporting Standards (CHEERS; for health economic evaluation)^43^, and Standards for Quality Improvement Reporting Excellence (SQUIRE; for implementation aspects)^44^.

## Discussion

POPS-SUp is a large-scale implementation study of CGA-based POPS services and is anticipated to provide definitive, practice-changing results. It has been fully co-designed with patient, public, and professional involvement and engagement. POPS-SUp uses a hybrid implementation–effectiveness study design to examine the impact of two interventions: a trimodal implementation strategy to support implementation of POPS services and a CGA- and optimization-based perioperative service (POPS).

Randomized clinical trials (RCT) are usually considered to be the standard design because they allow causal interpretation of the estimated intervention effect, but rely on being able to randomize individuals or clusters of individuals to an intervention or control group. Such an RCT approach was not appropriate for POPS-SUp due to the lack of an index disease or condition with which to randomize individual patients, the need to evaluate implementation alongside clinical effectiveness, and the scale (time and resource) required to minimize confounding and bias. Furthermore, it is now accepted that implementation processes and learning do not happen linearly at the end of a trial or evaluation period, but are better viewed as a non-linear context-contingent process, whereby implementation is a significant factor in influencing effectiveness and vice versa. Cognizant of this literature and following co-production with patients, patient-facing organizations, clinicians, academics, managers, and policymakers, POPS-SUp will use a hybrid implementation–effectiveness study design, with an embedded economic and process evaluation, to ensure simultaneous and efficient investigation. The pace of evaluation was also pertinent due to challenges in delivering elective recovery following the COVID-19 pandemic.

POPS-SUp will use an ITS design, acknowledged to be the standard approach where RCTs are not feasible^20^. The use of an ITS design will facilitate real-world data collection before and after implementation to meaningfully answer the research question relating to both implementation and clinical effectiveness and cost-effectiveness. Furthermore, this approach will limit selection bias and confounding due to between-site differences, because the sites will provide internal comparison, thus avoiding bias related to heterogeneous patient populations and differences in context and workforce across sites. This approach will allow standardization of the design and delivery of the complex intervention while allowing adaptation to the local context, maintaining fidelity. This will help overcome issues with studying the impact of an intervention during time of co-existent service or policy change. Finally, the use of an ITS has a sound theoretical basis, underpinned by Medical Research Council guidelines and NIHR frameworks for developing and evaluating complex interventions in situations where RCTs cannot provide clinically meaningful data on both implementation and effectiveness in a timely manner^20^. Specifically, ITS studies allow mitigation of selection bias, internal comparison per site, evaluation of a standardized intervention allowing for local adaptation, and concurrent policy change^45^.

Similarly, the outcome measures were co-produced with patient, public, and professional advisory groups. Length of hospital stay was chosen as the clinical co-primary outcome measure for several reasons. First, there is a direct relationship between CGA intervention and length of hospital stay through effects on postoperative complications and social factors, thus mitigating delay to discharge. Second, a reduction in length of hospital stay is a major determinant of hospital costs per episode of care. Third, patient and public partners reiterated that spending as few days in hospital as possible was very important to them and/or their families. Fourth, the length of hospital stay is routinely collected by hospitals, ensuring feasibility and minimizing bias. Reach was chosen as the implementation co-primary outcome because it is not specific to the type of surgery or patient population, it is an objective assessment minimizing bias, and it can be recorded from routinely collected clinical data. Extensive patient and public co-design provided strong advocacy for the use of routinely collected clinical data, without individual patient consent to minimize burden for patients and to avoid duplication, maximizing efficiency for healthcare professionals:

Why would we keep on spending money, that is so limited, on testing what has already been shown to work, not only here in the England but also in other countries. We need to get on with helping other hospitals set up POPS and that will tell us if it works. (Anonymous)

The anticipation is that POPS-SUp will have impact at individual (patient and clinician), organizational, and policy levels in the perioperative setting, but also with potential application to other clinical settings.

## Supplementary Material

zraf063_Supplementary_Data

## Data Availability

No new data were created or analysed for this article. Data sharing is not applicable.
