# Supplementary material for: Perioperative Medicine for Older People Undergoing Surgery Scale Up (POPS-SUp): study protocol
Source: BJS Open. 2025 Nov 19;9(6):zraf063. doi: 10.1093/bjsopen/zraf063 (PMC12628784; doi:10.1093/bjsopen/zraf063)
Supplement: zraf063_Supplementary_Data [file zraf063_supplementary_data.docx]

**Title**

Perioperative medicine for Older People undergoing Surgery Scale Up (POPS-SUp): Study Protocol

**Authors**

Jugdeep K Dhesi*^1,2^, Judith SL Partridge*^1,2^, Bridget C Strasser^1,2^, Lindsay Bearne^3^, Nathan Hall^4^, Andrew Healey^5^, John SM Houghton^6^, Laura Magill^7^, Bijan Modarai^8^, Iain K Moppett^9^, Lawrence Mudford^10^, John Norrie^11^, Rupert M Pearse^12^, Thomas Pinkney^7^, Athanasios Saratzis^6^, Robert Sayers^6^, Cecilia Vindrola-Padros^13^, Justin Waring^14^

*Co-first authors

1. Perioperative medicine for Older People undergoing Surgery (POPS), Guy’s and St Thomas’ NHS Foundation Trust
2. Ageing Research, Population Health Sciences, Life Sciences and Medicine, King’s College London
3. Population Health Research Institute, City St Georges, University of London
4. NHS England
5. Health Services and Population Research Department, Institute of Psychiatry, Psychology and Neuroscience, King’s College London
6. University of Leicester, Department of Cardiovascular Sciences, NIHR Leicester Biomedical Research Centre
7. Birmingham Centre for Observational and Prospective Studies (BiCOPS), University of Birmingham
8. Cardiovascular and Metabolic Medicine & Sciences, British Heart Foundation Centre, Life Sciences and Medicine, King’s College London
9. University of Nottingham & Nottingham University Hospitals NHS Trust
10. Centre for Perioperative Care (CPOC)
11. School of Medicine, Dentistry and Biomedical Sciences, Queen’s University, Belfast
12. Critical Care and Perioperative Medicine Research Group, Queen Mary University of London
13. Department or Targeted Intervention, University College London
14. School of Social Sciences, Loughborough University

**Corresponding author**

Dr Bridget Strasser

Guy's Hospital, Great Maze Pond, London, SE1 9RT

[Bridget.strasser@gstt.nhs.uk](mailto:Bridget.strasser@gstt.nhs.uk)

ORCID ID: [0009-0008-5461-5330](http://orcid.org/0009-0008-5461-5330)

**Supplementary Materials - Index**

| **Supplementary figures and tables** | *Page* |
| --- | --- |
| Table 1: The perioperative medicine for older people undergoing surgery (POPS) logic model (18) | *3-8* |
| Table 2: Routinely collected clinical data | *9-12* |
| **References** | *13-15* |
|  | *`* |

**Supplementary table 1:**

Supplementary table 1: The perioperative medicine for older people undergoing surgery (POPS) logic model (18)

| **Inputs** | **Core components** | **Mechanism (process changes)** | **Contextual factors (which enable or hinder implementation)** | **Short-term outcomes** | **Long-term outcomes** | **Wider impact of POPS service** |
| --- | --- | --- | --- | --- | --- | --- |
| **MDT POPS team** Consultant with expertise in CGA in perioperative setting CNS OT Administrator | Deliver preoperative CGA and optimisation through multidisciplinary working. | Preoperative CGA and optimisation outpatient clinic. Effective screening and referral criteria. Evidence-based perioperative medicine. ‘Hands on’ delivery of clinical optimisation plan. | ‘Buy-in’ from board, surgeons, anaesthetists and other key stakeholders. Skills and engagement of medical and AHP staff equipped to deliver CGA and optimisation in the perioperative period. IT infrastructure, technical support and physical space. Funding. | Improved quantification of perioperative risk. Improved shared decision making. Improved identification of underlying medical issues. Improved medical optimisation. Improved identification and management of barriers to early discharge. Reduced variation in patient care. | **Patient outcomes** Embedding of multidisciplinary and patient-centred shared decision making in clinical practice. Provision of timely surgical care. Avoidance of inappropriate procedures. Reduced postoperative morbidity and harm. Improved patient reported outcomes (short- and long-term). **Service outcomes** Improved quality of care. Reduced length of stay. Reduced same day cancellation of surgery. Reduced readmissions. Reduced financial cost. **Implementation outcomes** Acceptability of service to providers, patients and carers. Maintenance of fidelity while adapting service to the local context. Development of ‘big data’ through collation of audit and quality improvement work at POPS sites. Development of a workforce equipped to deliver perioperative medicine services for complex older patients. | Improved health literacy with consequently improved patient navigation of health services. Integrated healthcare. Improved health outcomes. Development of evidence base in perioperative medicine through big data. Influence on policy and funding streams for perioperative medicine services. |
| **Other key stakeholders** Trust executive/board Surgeons Anaesthetists Other AHPs Surgical pathway coordinators Evaluator Patients and relatives | Provide postoperative CGA on the surgical ward. | CGA and optimisation conducted using scheduled ward rounds, board rounds, multidisciplinary team meetings. Proactive communication between healthcare professionals, patients and families. | ‘Buy-in’ from ward teams. Staff attitudes and behaviours. Skills and engagement of medical and AHP staff equipped to deliver CGA and optimisation in the perioperative period. IT infrastructure, technical support and physical space. Funding. | Fewer acute postoperative complications. Improved early identification of postoperative complications with standardised management. Safe and effective discharge planning. |  |  |
| **Resources** Funding Clinic space Office space Equipment Supporting pro formas (in paper or electronic format) Clinical reference guide Clinical guidelines/protocols | Ensure ownership of patient care. | Fostering an environment that promotes shared decision making with the patient at the centre. | Professional jurisdictions, norms and codes of behaviour. Readiness for change within the clinical team and the organisation (open to new ways of delivering collaborative patient-centred care). | Reduced number of specialist referrals. Single point of access: improved communication with patient and primary care to facilitate patient navigation of perioperative pathway. Improved shared decision making. Improved delivery of holistic care, with focus on perioperative and longer-term outcomes. |  |  |
| **Education resources** Doctor/CNS curriculum / competency framework Teaching schedules Teaching materials (eg slide-packs) | Facilitate proactive liaison with other teams. | Joint ward rounds, board rounds, clinic consults between surgery, medicine and anaesthetics. Joint audit meetings / teaching sessions between surgery, medicine and anaesthetics. | Buy-in from all stakeholders. Promotion of collaborative working (on individual, team and organisational level). Trusted informal and formal peer review and accountability with willingness to engage with feedback processes. Avoidance of silo working and poor communication (whether due to IT or other systems). | Informed and therefore improved clinical decision making. Reduced silo culture. Positive influence on behaviours and attitudes of junior medical and nursing staff. |  |  |
|  | Provide education and training to POPS team and key stakeholders. | Predefined curriculum. Structured teaching programme. | Scheduled protected teaching sessions. | An ‘upskilled’ workforce able to provide care for complex older patients. Improved knowledge and skills in perioperative medicine (doctors and AHPs). Creation of teaching and mentoring opportunities. |  |  |
|  | Establish governance structure and evaluation programme. | Structured quality improvement programme underpinned by robust clinical governance meetings. | Support from informatics. Access to expertise in quality improvement. | Ensure fidelity to the POPS model of care. Reduced unwarranted variation in patient care. Improved quality of care. Development of an evidence base in issues relevant to complex older surgical patients. |  |  |

AHPs = allied health professional; CGA = comprehensive geriatric assessment; CNS = clinical nurse specialist; MDT = multidisciplinary team; OT = occupational therapist; POPS = perioperative medicine for older people undergoing surgery.

Supplementary table 2: Routinely collected clinical data

| Outcomes | Data | Timing of collection | Who will collect | Consent |
| --- | --- | --- | --- | --- |
| Primary implementation | Reach | Weekly | Local clinical and research team | No |
| Primary clinical | Length of stay (days) | Weekly | Local clinical and research team | No |
| Secondary implementation | Fidelity to clinical components of perioperative CGA | Collected once in month 3 (preimplementation phase), once in month 9 (implementation phase) and once in month 12 (postimplementation phase) | Research team | Yes (written consent from staff members) |
|  | Fidelity to core components of POPS services | Collected once in month 3 (preimplementation phase), once in month 9 (implementation phase) and once in month 12 (postimplementation phase) | Research team | Yes (written consent from staff members) |
|  | Acceptability | Collected once in month 3 (preimplementation phase), once in month 9 (implementation phase) and once in month 12 (postimplementation phase) | Research team | Yes (written consent from staff members) |
|  | Feasibility | Collected once in month 3 (preimplementation phase), once in month 9 (implementation phase) and once in month 12 (postimplementation phase) | Research team | Yes (written consent from staff members) |
| Secondary clinical | Age | Once | Local clinical and research team | No |
|  | Gender | Once | Local clinical and research team | No |
|  | Clinical frailty scale | Once | Local clinical and research team | No |
|  | Number of regular medications | Once | Local clinical and research team | No |
|  | Count of comorbidities | Once | Local clinical and research teams | No |
|  | 30-day readmission | Weekly | Local clinical and research team | No |
|  | Postoperative delirium (documented new confusion or delirium, documented 4AT or CAM score) | Weekly | Local clinical and research team | No |
|  | Acute coronary syndrome, MI (troponin rise/ECG changes with treatment for ACS/MI) | Weekly | Local clinical and research team | No |
|  | Cardiac failure (BNP/echo with treatment for heart failure) | Weekly | Local clinical and research team | No |
|  | Arrythmia (ECG/cardiac monitor with treatment for arrythmia) | Weekly | Local clinical and research team | No |
|  | Pneumonia (symptoms +/- raised inflammatory markers +/- radiological change with treatment for pneumonia) | Weekly | Local clinical and research team | No |
|  | Acute kidney injury (increase in serum creatinine by ≥ 0.3 mg/dl (≥ 26.5 µmol/l) within 48 hours; or increase in serum creatinine to ≥ 1.5 times baseline, which is known or presumed to have occurred within the prior 7 days) | Weekly | Local clinical and research team | No |
|  | Surgical site infection (clinical evidence or positive swab with treatment for infection) | Weekly | Local clinical and research team | No |
|  | Urinary tract infection (dysuria/supra public pain / new unexplained delirium with positive urine culture with treatment for UTI) | Weekly | Local clinical and research team | No |
|  | Catheter issue (failed trial without catheter/ traumatic haematuria) | Weekly | Local clinical and research team | No |
|  | Fall | Weekly | Local clinical and research team | No |
|  | Other | Weekly | Local clinical and research team | No |
|  | Same day cancellation | Weekly | Local clinical and research team | No |
|  | Return to preoperative place of residence | Weekly | Local clinical and research team | No |
|  | Operative or non-operative management | Weekly | Local clinical and research team | No |
|  | Was the initially suggested procedure undertaken or did the patient undergo a different or no procedure? | Weekly | Local clinical and research team | No |
|  | Clinician defined, ‘medically fit for discharge’ | Weekly | Local clinical and research team | No |
|  | Health economic metrics for opportunity cost  Investigations  Referrals to other teams  Level 2/3 care. | Collected once at end of month 3 (preimplementation phase), once at end of month 9 (implementation phase) and once at end of month 12 (postimplementation phase) | Local clinical and research team | No |
|  | Days alive and out of hospital 90 days | 12 months | Research team | No |
|  | 90 day and 12 month mortality | 12 months | Research team | No |
|  | SDM Q9* | Pre, during and post implementation | Research team | Yes |
|  | DRS* | Pre, during and post implementation | Research team | Yes |

*

-Shared decision making (SDMQ9) (collected in a purposively sampled consented subgroup of patients) to be collected in the preimplementation and in postimplementation phase

-Decisional regret (Decision Regret Scale) (collected in a purposively sampled consented subgroup of patients) to be collected in the preimplementation and in postimplementation phase (6 patients per site to be consented in each of the pre and post implementation phases across 18 sites. Purposive sampling to include elective/emergency, surgery/no surgery and LoS</> 5 days. Applies to SDMQ9 and DRS. Estimated total 216 patients)

**References**

1. Fowler AJ, Abbott TEF, Prowle J, Pearse RM. Age of patients undergoing surgery. British Journal of Surgery. 2019 Jul 1;106(8):1012–8.

2. Parmar KL, Law J, Carter B, Hewitt J, Boyle JM, Casey P, et al. Frailty in Older Patients Undergoing Emergency Laparotomy. Ann Surg. 2021 Apr;273(4):709–18.

3. Fowler AJ, Wahedally MAH, Abbott TEF, Smuk M, Prowle JR, Pearse RM, et al. Death after surgery among patients with chronic disease: prospective study of routinely collected data in the English NHS. Br J Anaesth. 2022 Feb;128(2):333–42.

4. Hamel MB, Henderson WG, Khuri SF, Daley J. Surgical Outcomes for Patients Aged 80 and Older: Morbidity and Mortality from Major Noncardiac Surgery. J Am Geriatr Soc. 2005 Mar 24;53(3):424–9.

5. BGS. Comprehensive Geriatric Assessment Toolkit for Primary Care Practitioners. 2015.

6. Ellis G, Gardner M, Tsiachristas A, Langhorne P, Burke O, Harwood RH, et al. Comprehensive geriatric assessment for older adults admitted to hospital. Cochrane Database of Systematic Reviews. 2017 Sep 12;2017(9).

7. Partridge JSL, Harari D, Martin FC, Peacock JL, Bell R, Mohammed A, et al. Randomized clinical trial of comprehensive geriatric assessment and optimization in vascular surgery. British Journal of Surgery. 2017 Apr 13;104(6):679–87.

8. Partridge JSL, Healey A, Modarai B, Harari D, Martin FC, Dhesi JK. Preoperative comprehensive geriatric assessment and optimisation prior to elective arterial vascular surgery: a health economic analysis. Age Ageing. 2021 Sep 11;50(5):1770–7.

9. Eamer G, Taheri A, Chen SS, Daviduck Q, Chambers T, Shi X, et al. Comprehensive geriatric assessment for older people admitted to a surgical service. Cochrane Database of Systematic Reviews. 2018 Jan 31;2018(3).

10. Aitken RM, Partridge JSL, Oliver CM, Murray D, Hare S, Lockwood S, et al. Older patients undergoing emergency laparotomy: observations from the National Emergency Laparotomy Audit (NELA) years 1–4. Age Ageing. 2020 Jul 1;49(4):656–63.

11. Centre of Perioperative Care. Guideline for Perioperative Care for People Living with Frailty. 2021.

12. The Royal College of Surgeons of England and Department of Health. The Higher Risk General Surgical Patient. 2021.

13. National Emergency Laparotomy Audit. National Emergency Laparotomy Audit.

14. Joughin AL, Partridge JSL, O’Halloran T, Dhesi JK. Where are we now in perioperative medicine? Results from a repeated UK survey of geriatric medicine delivered services for older people. Age Ageing. 2019 May 1;48(3):458–62.

15. NHS England, Getting it right first time. Anaesthesia and Perioperative Medicine [Internet]. 2021 [cited 2024 Dec 3]. Available from: https://gettingitrightfirsttime.co.uk/medical_specialties/apom/

16. Horton T, Illingworth J, Warburton W. The Spread Challenge. 2018 Sep.

17. Fulop NJ, Ramsay AIG, Perry C, Boaden RJ, McKevitt C, Rudd AG, et al. Explaining outcomes in major system change: a qualitative study of implementing centralised acute stroke services in two large metropolitan regions in England. Implementation Science. 2015 Dec 3;11(1):80.

18. Jasper E V, Dhesi JK, Partridge JS, Sadler E, Sevdalis N. Scaling up perioperative medicine for older people undergoing surgery (POPS) services; use of a logic model approach. Clinical Medicine. 2019 Nov;19(6):478–84.

19. Kriston L, Scholl I, Hölzel L, Simon D, Loh A, Härter M. The 9-item Shared Decision Making Questionnaire (SDM-Q-9). Development and psychometric properties in a primary care sample. Patient Educ Couns. 2010;80(1):94–9.

20. Brehaut JC, O’Connor AM, Wood TJ, Hack TF, Siminoff L, Gordon E, et al. Validation of a Decision Regret Scale. Medical Decision Making. 2003 Jul 1;23(4):281–92.

21. Cruz M, Bender M, Ombao H. A robust interrupted time series model for analyzing complex health care intervention data. Stat Med. 2017 Dec 20;36(29):4660–76.

22. Lopez Bernal J, Cummins S, Gasparrini A. The use of controls in interrupted time series studies of public health interventions. Int J Epidemiol. 2018 Dec 1;47(6):2082–93.

23. Lopez Bernal J, Soumerai S, Gasparrini A. A methodological framework for model selection in interrupted time series studies. J Clin Epidemiol. 2018 Nov;103:82–91.

24. European Medicines Agency. ICH E9 (R1) addendum on estimands and sentitivity analysis in clinical trails to the guideline on statistical principles for clinical trials. 2020.

25. Kahan BC, Hindley J, Edwards M, Cro S, Morris TP. The estimands framework: a primer on the ICH E9(R1) addendum. BMJ. 2024 Jan 23;e076316.

26. Turner SL, Karahalios A, Forbes AB, Taljaard M, Grimshaw JM, Cheng AC, et al. Design characteristics and statistical methods used in interrupted time series studies evaluating public health interventions: a review. J Clin Epidemiol. 2020 Jun;122:1–11.

27. Turner SL, Karahalios A, Forbes AB, Taljaard M, Grimshaw JM, McKenzie JE. Comparison of six statistical methods for interrupted time series studies: empirical evaluation of 190 published series. BMC Med Res Methodol. 2021 Dec 26;21(1):134.

28. Little RJ, D’Agostino R, Cohen ML, Dickersin K, Emerson SS, Farrar JT, et al. The Prevention and Treatment of Missing Data in Clinical Trials. New England Journal of Medicine. 2012 Oct 4;367(14):1355–60.

29. van Buuren S. Flexible Imputation of Missing Data. 2018.

30. Molenberghs G, Kenward MG. Missing Data in Clinical Studies. Wiley; 2007.

31. Zhang F, Wagner AK, Ross-Degnan D. Simulation-based power calculation for designing interrupted time series analyses of health policy interventions. J Clin Epidemiol. 2011 Nov;64(11):1252–61.

32. Hawley S, Ali MS, Berencsi K, Judge A, Prieto-Alhambra D. <p>Sample size and power considerations for ordinary least squares interrupted time series analysis: a simulation study</p>. Clin Epidemiol. 2019 Feb;Volume 11:197–205.

33. SAS Institute Inc. SAS for Windows.

34. R foundation for Statistical Computing. A Language and Environment for Statistical Computing. 2024.

35. StataCorp. Stata Statistical Software: Release 18. 2023.

36. Drummond M, Sculpher M, Claxton K, Stoddart G, Torrance G. Methods for the Economic Evaluation of Health Care Programmes. Fourth. Oxford University Press; 2015.

37. National Institute for Health and Care Excellence. Guide to the methods of technology appraisal 2013. 2013 Apr.

38. Vindrola-Padros C, Chisnall G, Cooper S, Dowrick A, Djellouli N, Symmons SM, et al. Carrying Out Rapid Qualitative Research During a Pandemic: Emerging Lessons From COVID-19. Qual Health Res. 2020 Dec 31;30(14):2192–204.

39. Gale NK, Heath G, Cameron E, Rashid S, Redwood S. Using the framework method for the analysis of qualitative data in multi-disciplinary health research. BMC Med Res Methodol. 2013 Sep 18;13(1):117.

40. Damschroder LJ, Reardon CM, Opra Widerquist MA, Lowery J. Conceptualizing outcomes for use with the Consolidated Framework for Implementation Research (CFIR): the CFIR Outcomes Addendum. Implementation Science. 2022 Dec 22;17(1):7.

41. NIHR. Improving inclusion of under-served groups in clinical research: Guidance from INCLUDE project [Internet]. 2020 Aug [cited 2024 Nov 14]. Available from: NIHR (2020) Improving inclusion of under-served groups in clinical research: Guidance from the NIHR-INCLUDE project. UK: NIHR. Available at: www.nihr.ac.uk/documents/improving-inclusion-of-under-served-groups-in-clinical-research-guidance-from-include-project/25435

42. Chan AW, Tetzlaff JM, Gotzsche PC, Altman DG, Mann H, Berlin JA, et al. SPIRIT 2013 explanation and elaboration: guidance for protocols of clinical trials. BMJ. 2013 Jan 9;346(jan08 15):e7586–e7586.

43. Husereau D, Drummond M, Augustovski F, de Bekker-Grob E, Briggs AH, Carswell C, et al. Consolidated Health Economic Evaluation Reporting Standards 2022 (CHEERS 2022) statement: updated reporting guidance for health economic evaluations. BMC Med. 2022 Dec 12;20(1):23.

44. Ogrinc G, Davies L, Goodman D, Batalden P, Davidoff F, Stevens D. SQUIRE 2.0 ( *Standards for QUality Improvement Reporting Excellence)* : revised publication guidelines from a detailed consensus process. BMJ Qual Saf. 2016 Dec;25(12):986–92.

45. Handley MA, Lyles CR, McCulloch C, Cattamanchi A. Selecting and Improving Quasi-Experimental Designs in Effectiveness and Implementation Research. Annu Rev Public Health. 2018 Apr 1;39(1):5–25.
